# Early and precocious puberty during the COVID-19 pandemic

**DOI:** 10.3389/fendo.2022.1107911

**Published:** 2023-01-09

**Authors:** Sara Prosperi, Francesco Chiarelli

**Affiliations:** Department of Pediatrics, University of Chieti, Chieti, Italy

**Keywords:** precocious puberty, early puberty, puberty, secular trend, GnRH, COVID-19, BMI, lifestyle

## Abstract

During the year 2020, the COVID-19 pandemic rapidly became a severe health emergency worldwide. In order to contrast the spread of the novel SARS-CoV-2, many countries implemented extraordinary restrictive measures, such as a strict lockdown and school closures. The pandemic had a great impact on children and adolescents’ daily life, leading to a much more sedentary lifestyle, to larger use of electronic devices and to an increase in stress-related symptoms. These conspicuous changes acted as disruptors of children’s normal development. Since the beginning of the pandemic, many studies reported an increase in the number of precocious puberty cases as well as a faster progression rate of puberty itself, if compared to the pre-pandemic years. In this review, our aim was to evaluate the incidence of new cases of early and precocious puberty during the COVID-19 pandemic, analyzing variations in the timing of puberty and in pubertal progression rate, and to investigate the role of environmental and lifestyle factors during the pandemic in modulating the physiopathology of pubertal development. While a direct effect of SARS-CoV-2 infection remains, at the moment, a remote hypothesis, both physical and psychological factors related to the pandemic seem to have a role in triggering GnRH pulsatile secretion leading to earlier pubertal onset. It is indeed important to stress the need to clarify the exact role of COVID-19 in early pubertal onset comparing data from all over the world; long-term comprehensive studies are also pivotal to explain whether this phenomenon will continue while we resume pre-pandemic habits.

## 1 Introduction

During the first months of the COVID-19 pandemic, many governments implemented social distancing measures in order to contain viral diffusion; national lockdowns were the strictest measures. School closure and stay-at-home policy resulted in increased psychological stress (1), in the overuse of electronic devices and into a more sedentary lifestyle. Increases in body weight and BMI were reported among children and adolescents (2).

Since the beginning of 2020, several pediatric endocrinology centers observed an increase in referrals for suspected precocious puberty and in the number of central precocious puberty (CPP) and rapidly progressive puberty cases, mainly in girls. Many of those children needed to start therapy with GnRH analogs.

This new trend was first reported in November 2020 in Meyer Children’s University Hospital, Florence (3); many reports promptly came from other countries all over the world.

In the following paragraphs, after describing the mechanisms underlying pubertal development and the main causes of central and peripheral precocious puberty, the results of these studies will be examined to assess the influence of many aspects potentially linked with precocious puberty and rapidly progressive puberty. Both direct effects of SARS-CoV-2 infection and physical and psychological changes related to the pandemic will be investigated as potential underlying causes.

## 2 Puberty

Great advantages in understanding the physiology of puberty have been made in the last decades. Puberty can be defined as the transition period between childhood and adulthood. Development of secondary sexual characters and gonadal maturation happen during this phase, thus leading the individual to the achievement of reproductive capacity (4). Puberty is controlled by the hypothalamic-pituitary-gonadal axis.

A transient activation of the hypothalamic-pituitary-gonadal axis happens however from the first week of life and achieves its peak during the first 3-6 months of life leading to high gonadotropin and sex steroid levels. This postnatal activation of the axis, called “minipuberty”, allows for sexual organs maturation in both boys and girls (5). The transient rise in estradiol and testosterone levels is generally not followed by clinically visible changes. In rare cases, it may lead to clinically visible signs such as vaginal bleeding and transient breast budding in girls or palpable testicular growth and development of pubic hair in boys. Minipuberty grants the initial development of genital organs in both males and females, assessing the basis for their future sexual maturity and fertility. However, the exact role of this process needs to be further explored and light must be thrown on the exact mechanisms that make the hypothalamic-pituitary-gonadal axis turn itself off until puberty (5, 6).

As for the reactivation of the axis, it is probably due to a dense interaction between genetic, epigenetic, and neuroendocrine factors (7). Nutritional status, body weight, exercise and exposure to endocrine disruptors play a role too (8, 9).

Pubertal development is the result of increased secretion of hypothalamic gonadotropin-releasing hormone (GnRH), gonadotrophins and, subsequently, gonadal sex steroids. Gonadotropins (luteinizing hormone, LH, and follicle-stimulating hormone, FSH) are released in a pulsatile way from the pituitary gland as a response to GnRH stimulation. Studies demonstrate that what increases at puberty is GnRH pulse amplitude rather than its frequency (10, 11). Particularly, what triggers pubertal onset by stimulating GnRH pulse and gonadotropin secretion are the hypothalamic kisspeptin neurons, located in the arcuate nucleus of the hypothalamus (12). Authors demonstrated that mutations in GPR54, a G-protein coupled receptor for kisspeptin, cause hypogonadotropic hypogonadism and alter normal gonadotropin secretion and normal pubertal physiology. Mutations at different sites of the KISS1 gene, the gene encoding for kisspeptin, lead to pubertal absence or pubertal delay too (13, 14).

After the increase in amplitude of GnRH pulse, the rise in LH and FSH levels enhances the production of sex hormones by the gonads. Sex hormones, mainly estrogens, also mediate effects on growth rate, central nervous system functioning, bone mineralization (15).

Although there can be variations in the timing and tempo of puberty in boys and girls, the underlying mechanisms are the same. To assess pubertal development, physicians use detectable changes in secondary sexual characteristics to represent the degree of pubertal maturation. These modifications, described by Marshall and Tanner (16, 17), concern the degree of physical maturation of breasts and pubic hair in girls and of genitals and pubic hair in boys. The progression of these physical characteristics is described in five maturational stages, going from prepubertal stage to the achievement of full sexual maturity.

In females puberty usually starts between age 8 and age 13. Even though puberty pattern can vary, in most cases the first event is the thelarche (the onset of secondary breast development, Tanner stage B2), followed by pubarche (the first appearance of pubic and axillary hair). In the meanwhile there is an increase in linear growth that reaches its peak in stage 4. At this point, the menarche can happen. In some studies, it is debated if pubarche without breast development may represent, in girls, the true onset signal of pubertal development (18). In males, puberty starts between age 9 and age 14. The first marker of pubertal onset is a testicular volume size ≥4 mL by the Prader orchidometer (Tanner stage G2), followed by the growth of pubic and axillary hair and by the growth of the penis. During Tanner Stage 3 males usually undergo peak height velocity (19).

## 3 Precocious puberty

Precocious puberty is traditionally defined as the appearance of secondary sex characters before 8 years of age in girls and 9 years of age in boys (20). Precocious puberty needs to be differentiated from incomplete variants of puberty such as premature thelarche, premature adrenarche and premature menarche (21). Early puberty can be defined by the presence of secondary sexual characteristics between age 8 to 10 years in girls and 9 to 11 years in boys (22, 23) and is usually a condition that does not require therapy as it is often compensated by a longer duration of puberty itself and final adult height is normal (24, 25).

Based on the nature of its etiology, precocious puberty can be defined central or peripheral.

CPP derives from earlier activation and maturation of the hypothalamic-pituitary-gonadal axis. CPP happens through the same stages as normal pubertal development and is a disease with female preponderance. While the idiopathic etiology is the most common cause in girls, underlying tumors or injuries of the central nervous system (CNS) are more common in boys (26, 27). Recently, mutations in the kisspeptin family have been identified in cases of CPP (26). The most relevant conditions associated with the development of CPP are:

- CNS tumors;
- CNS injuries;
- Genetic variants and syndromes;
- Familial forms of CPP.

Peripheral precocious puberty (PPP) is less frequent than CPP and results from sex steroid exposure without the activation of GnRH pulsatile secretion. PPP can be acquired or congenital. Some important causes of PPP are (28–30):

- Congenital adrenal hyperplasia (CAH);
- McCune Albright syndrome;
- Familial male-limited precocious puberty (FMPP or testotoxicosis);
- Sex steroid secreting tumors;
- Exogenous exposure to sex steroids;
- Van Wyk Grumbach syndrome (hypothyroidism, precocious puberty, ovarian cysts).

In CPP, treatment with GnRH agonist is considered safe, without significant adverse effects, and can be administered based on the progression of puberty and on the age of the child to stop the development of secondary sex characteristics and to preserve final adult height. Late onset of treatment (especially in girls aged more than 6), advanced bone age, shorter duration of treatment are some of the factors linked with less success in preserving adult height (31).

In PPP the clinical presentation and the treatment of choice depend on the underlying cause. The primary purpose of therapy in PPP is to remove the extra source of sex steroids thus restoring prepubertal values of sex hormones (32).

Precocious puberty has been, in the past decades, a matter of concern as the decline in age at puberty has been associated in both boys and girls with several health risks and adverse behaviors. Changes in pubertal timing are related to rapid growth and reduced final adult height (33) and to a higher risk of developing characteristics of metabolic syndrome (34). Precocious puberty has been also linked with increased rate of reproductive tract cancers in adult life such as breast cancer (35). From a behavioral perspective, precocious puberty and rapid pubertal progression have been linked to more risky behaviors, earlier sexual activity, low self-esteem, psychosocial vulnerability (36, 37).

In the past decades a secular trend in puberty was observed and the average age at menarche decreased from 17 years in the mid-19^th^ century to approximately 13 years in the mid-20^th^ century (38–40). More recently the general decline in age at puberty has been accompanied by a simultaneous increase in number of referrals for precocious puberty.

A study conducted on more than 17000 girls aged 3 to 12 years in 1997, the Pediatric Research in Office Settings (PROS) study, showed a decline in the age of breast and pubic hair development. As a reply to this research, in 1999 the Lawson Wilkins Pediatric Endocrine Society proposed new guidelines to define precocious puberty (41), suggesting that girls had to be evaluated if breast or pubic hair development occurred before 7 years of age (in white girls) or 6 years of age (in African American girls). No changes were suggested for the evaluation of precocious puberty in boys as no secular trend was noticed in boys (40). Nonetheless some authors suggested a bias in the PROS study, as the population selected was not a random sample (42).

After the PROS study, European (43–45) and American (46) studies confirmed a decline in age at puberty in girls and physicians questioned the reasons behind this phenomenon. Although pubertal timing is mostly determined by genetic factors, it has been supposed that the environment has a role in causing earlier onset of puberty. There is evidence, in females, of a link between BMI, hyperinsulinemia, insulin resistance and early onset of puberty, but not always pubertal tempo (47). Endocrine disruptors can be defined as “environmental contaminants that perturb hormonal systems” (48). Recently the role of pre- and post-natal exposure to endocrine disrupting chemicals, a large proportion of which is represented by pesticides, has been discussed too as they seem to affect pubertal timing (8, 49, 50). Possible connections between increased screen time, blue light, sleep quality, melatonin levels and precocious puberty are debated too and further studies on these topics are required (51–53).

## 4 Precocious puberty during the COVID-19 pandemic

During the past two and a half years, multiple centers globally recorded an increase in CPP and rapidly progressive puberty diagnoses (**Table 1**).

**Table 1:** Summary of reviewed studies about the impact of COVID-19 on puberty.

Reference	Study type	Outcomes	Limitations
Stagi S et al.	Retrospective	-Increased incidence of newly diagnosed CPP and faster rate of pubertal progression in already diagnosed patients, during and after lockdown compared to previous years -Increased BMI -Augmented use of electronic devices	-Small sample size -The authors did not study the increase in calorie intake nor the decrease in physical activity
Verzani M et al.	Retrospective	-Increase in precocious puberty cases in girls in the first six months of the COVID-19 pandemic, compared to the same period of 2019 -No differences in the anthropometric parameters of the two groups	-Boys were not studied -Preliminary data; a potential link to specific pathogenetic factors was not studied
Umano GR et al.	Retrospective-prospective	-2.5-fold increase in CPP cases during lockdown if compared to the previous three years -Higher rates of sleep disturbances and later bedtime in new CPP cases -No differences in BMI and use of electronic devices -Increased estradiol, LH, and FSH levels in girls diagnosed after lockdown	-Boys were not studied -The link between sleep disturbances and CPP should be further investigated
Peinkhofer M et al.	Retrospective	-16% increase in CPP in girls during 2020 compared to the same period in 2019 -No increase in BMI SDS between early pubertal girls in 2020 if compared to 2019 -75% decrease in CPP in males	-Data collected from a single center
Chen Y et al.	Cross sectional	-Higher incidence of precocious puberty in 2020 than in 2016-2019 -Decreased serum concentrations of MKRN3 and ghrelin in the 2020 group -Decreased physical activities, increased food consumption and rapid weight gain in the pandemic group	-Small sample -Prolonged frozen storage of samples may have led to hormones degradation -As a cross sectional study, the link between MKRN3 and ghrelin was not studied
Fu D et al.	Retrospective	-Increased incidence of precocious puberty in girls in 2020 if compared to 2018-2019; -Potential link to prolonged use of electronic devices, changes in diet, decreased outdoor activities, vitamin D deficiency, increased BMI, genetic factors, endocrine disruptors.	-Small sample -Boys were not studied
Acinikli KY et al.	Retrospective	-3-fold increase in the number of CPP and rapidly progressive early puberty cases in the first 15 months of the pandemic if compared to the same period before -85% of CPP and 67% of rapidly progressive early puberty cases promptly started treatment with GnRH analogs -28% of CPP and 69% of rapidly progressive early puberty cases were obese or overweight and were diagnosed during the pandemic period	-Limited follow-up period -Past SARS-CoV-2 infection and many lifestyle changes potentially linked with the increased frequency of CPP were not investigated
Orman B et al.	Retrospective	-3-fold increase in the number of CPP and rapidly progressive puberty cases in three months of lockdown if compared to the same period from the previous year -Aarly admission age in patients who were given treatment for precocious puberty -Similar BMI SDS values between the two groups	-Data collected for three months only -Small sample size
Yesiltepe Mutlu G et al.	Retrospective	-Increase in precocious puberty and accelerated puberty cases during the pandemic -Increased need for pubertal suppression treatment at the time of presentation in the pandemic group -Lower age at presentation and lower age at pubertal onset in the pandemic group -Similar BMI SDS values between the two groups	-Lack of information on lifestyle changes
Mondkar SA et al.	Retrospective	-3-fold increase in the referrals for precocity during the lockdown period -Significant increase in referrals in girls (3.7 times) -Higher number of CPP diagnoses during the lockdown period -More advanced bone age in the pandemic group	-Lack of information on lifestyle changes

The first reports of this trend came from Italy. A retrospective study on patients referred to the Auxo-endocrinology and Pediatric Gynecology Unit of the Meyer Children’s University Hospital was published in November 2020. The Authors reported an increase in CPP cases during the first lockdown (March – July 2020); the number of new diagnoses was significantly higher if compared to the mean from the same period of time in the previous five years. They also collected data concerning the rate of pubertal progression and found that some patients who had previously received CPP diagnosis moved from a slowly progressive puberty to a rapidly progressive puberty during the first lockdown. As for the reason of this new trend, the first hypotheses concerned the increase in BMI and the augmented use of electronic devices during the first lockdown. In both CPP and rapidly progressive puberty group BMI increased significantly during the lockdown and families reported an augmented use of electronic devices (3).

A different Italian study conducted in the Endocrinology Unit of Bambino Gesù Children’s Hospital in Rome compared the first 2020 months with the same months in 2019 and described a significant increase in outpatient consultations for precocious puberty. In this case, however, no significant differences in BMI were reported between the first and the second group (54).

In the outpatient clinic of pediatric endocrinology of the University of Campania Luigi Vanvitelli, CPP incidence rate was 2.5-fold higher from April 2020 to April 2021 than from 2017 to 2020. While sleep disturbance was a frequently associated comorbidity, BMI was found to be similar between the two groups. CPP cases diagnosed during the pandemic had higher levels of LH, FSH and 17 beta estradiol than CPP cases diagnosed before the pandemic (55).

A significant increase in CPP diagnoses in girls in 2020 if compared to 2019 (+16%) was also found in Trieste, Italy. No increase in BMI was found in the 2020 group. Conversely, CPP diagnoses decreased in boys (-75%): the Authors traced this trend back to the fact that clinical findings such as testicular enlargement often went unnoticed and family pediatrician visits were missed during the first lockdown (56).

Similar results promptly came from research conducted all over the world. In a study among school-aged girls in Shanghai, the incidence rate of precocious puberty in 2020 was higher than that in 2016-2019; serum concentrations of MKRN3 (makorin ring finger protein 3, a negative regulator of GnRH) were lower and GnRH concentrations were higher in girls from the 2020 group. These girls had several risk factors related to lifestyle changes that happened during the first lockdown such as weight gain (the median value of weight gain was 2 kg in 6 months), augmented screen time, reduced physical activity (57). An analogous report came from a study involving 22 medical units from 13 cities in Henan Province, China. The Authors described a three-fold increase in the number of precocious puberty cases in girls in 2020 if compared to 2019. Weight and BMI were significantly higher in the 2020 group. Other potential risk factors that arose were augmented use of electronic devices, reduced physical activity, vitamin D deficiency, consumption of processed food (58).

In a report from Turkey, the Authors highlighted how the number of CPP and rapidly progressive early puberty diagnoses increased three times in the pandemic period (April 2020 – June 2021) if compared to the pre-pandemic period (January 2019 – March 2020). Among these girls, 85% of the ones diagnosed with CPP and 67% of the ones diagnosed with rapidly progressive early puberty needed to start treatment with GnRH analogs. Interestingly, the Authors noticed that 28% of CPP and 69% of rapidly progressive early puberty cases were obese or overweight and were diagnosed during the pandemic period (59).

However the correlation with an augmented BMI was not described in other Turkish reports. In the first one, the Authors reported an increase in both CPP cases and in rapidly progressive puberty during the first months of the pandemic, with earlier age at the diagnosis and at the beginning of treatment. The role of environmental factors was considered; BMI SDS (standard deviation score), however, was similar between the two groups (60). A different study conducted among several pediatric endocrinology clinics in Turkey reported an increase in precocious puberty and significantly accelerated puberty cases during the pandemic; the need for pubertal suppression treatment with GnRH analogs was augmented too. Comparing the two groups, age at presentation and age at pubertal onset were lower in the pandemic group than those in the pre-pandemic group but there was no significant difference between the BMI SDS values of the two groups (61).

Data from a tertiary level pediatric endocrinology center in Western India are consistent with data from other countries. The Authors compared a pre-lockdown and a lockdown group of boys and girls and showed a three-fold increase in the referrals for precocity. A higher increase was reported in girls. Mean weight and BMI were not significantly different between the pre-lockdown and the lockdown group. In most cases a rapidly progressive puberty was observed with a more advanced bone age (62).

Analyzing data from the past two and a half years, some limitations arise. Most studies considered a girls only population. Male precocious puberty is less common and more often linked to organic causes. However, the role of environmental factors on male pubertal timing is still not studied and understood enough.

Many studies had a retrospective nature and did not define how many patients had a confirmed SARS-CoV-2 infection thus preventing the Authors to assess a possible direct effect of COVID-19.

While nutritional status and BMI are known factors that affect pubertal timing, not all the studies conducted during the COVID-19 outbreak reported a correlation between precocious puberty and an augmented BMI. Furthermore, the potential risk factors for precocious puberty taken into account in the studies were not homogeneous.

On a side note, it has to be considered that some of the patients that received a diagnosis during the pandemic had pubertal onset prior to that time.

## 5 Direct effects of SARS-CoV-2 infection

Puberty is characterized by a broad range of physical and psychological changes necessary for the achievement of full reproductive maturity. These changes parallel modifications within the CNS (63): in a period in which the hypothalamus is so plastic, a possible direct effect of SARS-CoV-2 infection on hypothalamic cells has to be investigated.

Taking a step back, GnRH neurons in the hypothalamus share common embryonic origin with the olfactory bulb (OB): they develop outside the brain, in the olfactory placode, and then migrate to the hypothalamus during embryogenesis. Then, when this process is complete, GnRH neurons project their axons to the median eminence and from there they release GnRH (63). Some authors suggested a strong positive correlation between OB volume, pituitary length and precocious puberty (64).

Although there is no current proof of neuroinvasiveness of SARS-CoV-2 and neuroimaging during the acute infection is rare due to disease control and safety concerns, there is evidence for abnormal brain findings in autopsies and viral spread from the olfactory epithelium to the OB has been demonstrated (65). However there is currently no clue on what the dominant mechanisms in initiating puberty might be: direct OB inflammation (65), blood-brain barrier disruption (66) or cytokine storm (67).

Inflammatory cytokines may play a crucial role also stimulating N-methyl-D-aspartate (NMDA) receptors which promote GnRH pulsatile secretion through inputs from neurotransmitters such as glutamate (65, 68, 69).

## 6 Physical and psychosocial changes related to the pandemic

In the first months of the COVID-19 pandemic, when vaccines were still unavailable, containment measures such as lockdown and social distancing were effective in limiting viral transmission thus reducing case incidence and growth rate (70).

On the other hand, these measures had negative effects on both physical and psychological health in adults as well as in children and adolescents (71, 72). These new circumstances led to increased levels of stress and anxiety, greater rates of depression, and a feeling of helplessness (73, 74). Moreover, it has been noted how the COVID-19 pandemic will probably have long term adverse effects on children and adolescents’ health: hence the need for longitudinal, extended research on children and adolescents during and after the pandemic (75, 76).

Psychological factors such as fear and anxiety could have played a part in the outbreak of precocious puberty cases mediating an effect on CNS mediators such as NMDA (77) and glutamate (78). The rise of anxiety levels could have triggered the activation of GABA A receptors in prepubertal subjects, inducing the activation of stress pathways that are known to be typical of pubertal onset (79). Currently available data on changes in CNS mediators during the pandemic are nevertheless not enough to account for the rise of precocious puberty cases and further studies are required.

The main lifestyle changes regarded the reduction of both indoor and outdoor physical activities during the first pandemic wave (80, 81). This more sedentary routine, together with the worsening of eating habits, intensified the burden of childhood overweight and obesity. Many studies observed an increased consumption of total packaged snacks during the day as well as of sugary food, high calory food and processed meat (82–84). As a result, not only an increase in BMI was observed (85), but there were also serious alterations in glucose and insulin metabolism, specifically in already overweight and obese children: fasting glycemia, glucose, insulin excursion were significantly higher in children during the pandemic if compared to data from pre-pandemic children (86).

Childhood obesity has been associated with the secular trend of puberty anticipation, especially in girls, observed over the last few decades (87, 88). Some authors found that the precocious onset of pubertal development in these children might be related to the presence of higher leptin levels (89), which stimulate the production of sex hormones. The rapidity of weight gain itself may conduce to higher leptin and lower ghrelin levels. In a recent study conducted among Shanghai girls, the precocious puberty group was characterized by higher serum concentrations of GnRH and lower concentrations of ghrelin, confirming the important role of ghrelin in regulating pubertal mechanisms (57).

There is also a positive correlation between hyperinsulinemia, alone or in conjunction with adiposity, insulin resistance and precocious puberty (90, 91). It has been suggested that insulin resistance, in particular, may lead to an increased bioavailability of sex hormones by reducing the levels of sex hormones binding proteins (92). The increased levels of free sex hormones may trigger pubertal onset.

Though playing an important role in the rise in precocious puberty diagnoses during the pandemic, weight gain and BMI have probably acted together with other environmental factors.

As a result of the pandemic, screen time increased in children as well as in adults. In prepubertal age, particularly, screen time increased both due to online school lessons and to the reduction of social interactions and outdoor activities. Studies reported increases in both total and leisure screen time (93), with more hours spent watching TV and using smartphones and video games.

Another condition observed during the first months of the pandemic was a decline in vitamin D levels related to the reduction of time spent outdoors and of sun exposure because of the restrictions (94). Vitamin D deficiency has been linked to diabetes, obesity and autoimmune diseases. New studies show that vitamin D deficiency can also be associated with precocious puberty, particularly in girls (95, 96). Further research on this topic is needed to better clarify this potential link.

In the past decades, sleep changes as well as melatonin levels have been linked to pubertal development and, although studies on this topic are limited, data suggest that high melatonin levels in prepubertal age have an inhibitory effect on pubertal progression (97). In another study, the levels of 6-sulphatoxymelatonin were highest in the prepubertal group of girls and lowest in girls affected by precocious puberty (53). During the pandemic, an increased rate of sleep problems (assessed by personal perception and with scientifical instruments) was reported in families (98). In children and adolescents, sleep duration, bedtime and overall sleep quality got worse during the first lockdown, and that was probably due to both psychological factors and to increased usage of digital devices (99). It is still unknown if these changes could have played a role in the rise of precocious puberty cases.

The increase in the use of disposable items could have also contributed to a greater exposure to endocrine disrupting chemicals such as bisphenol A (BPA) and phthalate esters, used as additives in the making of plastic (100, 101). Several endocrine disrupting chemicals are known to influence the onset and the progression of puberty (102).

## 7 Conclusions

During the first phase of the COVID-19 pandemic, many governments applied social distancing measures and strict lockdowns to prevent the spread of the disease. As a result, changes in children’s physical and psychological health were recorded. An interesting finding was an increase not only in precocious puberty cases, but also in pubertal progression rate.

It is still not sure if SARS-CoV-2 infection can play a direct role in this event through direct OB inflammation, blood-brain barrier disruption or cytokine storm. There is instead more research on lockdown effects. Changes in lifestyle could likely have functioned as precocious puberty triggers. Changes in BMI, glucose and insulin metabolism, screen time, sleep quality, vitamin D levels, endocrine disruptors are all factors potentially linked to sex hormones modifications and early onset of puberty as well as faster pubertal progression rate (**Table 2**).

**Table 2:** – Possible causes of increased precocious puberty rate during the COVID-19 pandemic.

Direct SARS-CoV-2 infection effects	Direct olfactory bulb inflammation Blood-brain barrier disruption Cytokine storm
Physical and psychological changes related to the pandemic	Stress, fear, anxiety Reduction of physical activity and time spent outdoor Increased BMI Alterations in glucose and insulin metabolism Increased screen time and blue light exposure Poor sleep quality Vitamin D deficiency Exposure to endocrine disrupting chemicals

To further validate these hypotheses and better explain the exact role of SARS-CoV-2 infection and of the pandemic, more results from specialized centers from all over the world are expected in the near future. In the last analysis, two remaining questions arise. The first one regards parents’ role, as, maybe, the increase in precocious puberty referrals could be linked to closer monitoring of children during the pandemic. Parents working from home may have spent more time with their children and this may have helped in identifying in advance pubertal changes. The second one is about the duration of this phenomenon and whether it will continue or not while we resume pre-pandemic habits.

## Author contributions

The authors confirm contribution to the paper as follows: SP and FC contributed to conception and design of the review. SP drafted the manuscript. FC edited and revised the manuscript. Both authors contributed to the article and approved the submitted version.
